# A global dataset on species occurrences and functional traits of Schizothoracinae fish

**DOI:** 10.1038/s41597-024-03098-2

**Published:** 2024-03-06

**Authors:** Tingqi Du, Chengzhi Ding, Ke Yang, Jinnan Chen, Xingchen Liu, Wenna Lv, Liuyong Ding, Dekui He, Juan Tao

**Affiliations:** 1 Yunnan Key Laboratory of International Rivers and Transboundary Eco-Security, Yunnan University, Kunming, 650091 China; 2 Institute of International Rivers and Eco-security, Yunnan University, Kunming, 650091 China; 3 Institute of Yunnan Plateau Indigenous Fish, Kunming, 652115 China; 4 Ministry of Education Key Laboratory for Transboundary Eco-Security of Southwest, Kunming, 650500 China; 5 Institute of Hydrobiology, Chinese Academy of Sciences, Wuhan, 430072 China; 6 University of Chinese Academy of Sciences, Beijing, 100049 China

**Keywords:** Ichthyology, Conservation biology

## Abstract

The Schizothoracinae fish are a natural group of cyprinids widely distributed in rivers and lakes in the Qinghai-Tibetan Plateau (QTP) and adjacent regions. These fish parallelly evolved with the QTP uplift and are thus important for uncovering geological history, the paleoclimatic environment, and the mechanisms of functional adaptation to environmental change. However, a dataset including species occurrences and functional traits, which are essential for resolving the above issues and guiding relevant conservation, remains unavailable. To fill this gap, we systematically compiled a comprehensive dataset on species occurrences and functional traits of Schizothoracinae fish from our long-term field samplings and various sources (e.g., publications and online databases). The dataset includes 7,333 occurrence records and 3,204 records of 32 functional traits covering all the genera and species of Schizothoracinae fish (i.e., 12 genera and 125 species or subspecies). Sampling records spanned over 180 years. This dataset will serve as a valuable resource for future research on the evolution, historical biogeography, responses to environmental change, and conservation of the Schizothoracinae fish.

## Background & Summary

The Qinghai-Tibetan Plateau (QTP), which is renowned as the “Roof of the World,” “Third Pole,” and “Water Tower of Asia,” is the world’s largest high-elevation plateau with an average elevation of over 4,500 m and covering a 2,500,000 km^2^ area^1,2^. This region possesses about 46,000 glaciers and develops the major large rivers of Asia, such as the Yellow, Yangtze, Lancang-Mekong, Nu-Salween, Dulong-Irrawaddy, Yarlung Tsangpo-Brahmaputra, Ganges, and Indus rivers, providing water to over 20% of the global population^1,2^. The QTP uplift since the early Cenozoic has dramatically altered the Earth’s environment (e.g., separates the westerlies and forms Indian and Asian monsoons) and biodiversity distribution by engaging complex geologic, atmospheric, and hydrologic processes^3–6^. There are three major biodiversity hotspots with high species richness and many rare and endemic species surrounding the QTP^7^. These biota parallelly evolved with the environmental change caused by the QTP uplift and thus are a valuable source for uncovering the geological history, paleoclimatic environment, and mechanisms of functional adaptation to environmental change^8–11^. In addition, because the QTP is among the most sensitive areas to recent climate change, these organisms are also ideal for studying relevant biological responses, providing a scientific basis for predicting and mitigating the effects of climate change^12^. Among them, schizothoracine fish (Cyprinidae: Schizothoracinae) are the most representative taxon in aquatic ecosystems^13–16^.

The Schizothoracinae fish are widely distributed in rivers and lakes in the QTP and its surrounding areas^17^. They are the only natural group of Cyprinidae fish adapted to the extreme environmental conditions of the QTP. Currently, a total of over 100 Schizothoracinae species or subspecies belonging to 12 genera have been recorded^17,18^. These species greatly support regional fish diversity and wild fisheries and are important to maintaining the structure and function of relevant ecosystems^17^. Phylogenetically, these species were diverged from the primitive Barbinae fish through accumulating genetic and morphological traits adapted to environmental changes in response to QTP uplift^14^. Unlike terrestrial organisms that can rapidly disperse over long distances and in multiple directions on land, freshwater fish are strictly constrained in drainage systems, restricting their gene flow, and thus promoting local diversification and speciation. Therefore, genetic differences between different species and populations of schizothoracine fish have been suggested as suitable biological evidence for inferring the geologic history of the QTP uplifts and large river formations^14,19,20^. For example, the fossils of Schizothoracinae fish have been used to estimate the paleo-elevation of the QTP, and the results indicate that there have been large spatial and temporal differences in the uplift since the Oligocene^8,21^. Based on the degrees of morphological specialization (e.g., scales, pharyngeal teeth, and barbs) and distribution of modern Schizothoracinae fish, Cao and colleagues argued that the three evolutionary stages of them are closely related to the uplift processes of the QTP^14^. Accordingly, the Schizothoracinae fish can be grouped into three grades, including the primitive grade, the specialized grade, and the highly specialized grade. A molecular phylogeny of 24 Schizothoracinae species estimated that the average altitude of the QTP in the late Miocene should be between 2,750 and 3,750 m, providing a different perspective than sediment records^20^. In addition, studies have also shown that these fish are sensitive in response to recent climate change through changing growth and reproductive phenology^16,22^. The Schizothoracinae is one of the most threatened subfamilies in China, with 55% of species under threat^23^.

Species occurrences and functional trait information are fundamental to understanding biodiversity distribution patterns, predicting biological responses to environmental change, and promoting relevant conservation and management. This is because the functional trait composition and diversity of a community can reflect the characteristics and changes in the environment^24,25^. However, a dataset including such information for Schizothoracinae fish remains unavailable. Currently, their occurrence records and functional trait information are scattered in a wide range of sources (e.g., books, journal articles, master theses, doctoral dissertations, and online databases). The relevant knowledge held by most researchers and managers is outdated and mostly comes from surveys and published literature from the last century^14,18,26^. In addition, most of these data sources were written in Chinese, which poses a language barrier to interested non-Chinese researchers^27^. Although there are some large-scale databases (e.g., FishBase [https://www.fishbase.se/], Eschmeyer’s Catalog of Fishes [https://www.calacademy.org/scientists/projects/eschmeyers-catalog-of-fishes], and Global Biodiversity Information Facility [GBIF, https://www.gbif.org/]) related to freshwater fishes, Schizothoracinae fish are not targeted for consideration, and the included species and functional trait data are far from delicate and comprehensive. For example, a total of 78 Schizothoracinae species or subspecies were included in the most comprehensive global database of freshwater fish species occurrence at the drainage basin scale, without precise geographic coordinates or sampling time information^28^. The global database CESTES for metacommunity ecology, which integrates species, traits, environment, and space, does not include freshwater fish in Asia^29^. A Schizothoracinae-targeted database compiled the transcriptome data of 14 endemic species, but without precise sampling locations or functional trait information^30^. Therefore, it is urgently necessary to build a dataset containing species occurrences and functional traits of Schizothoracinae fish, given that the QTP has experienced more profound climate change and increasing anthropogenic disturbances^31,32^.

**Fig. 1:**
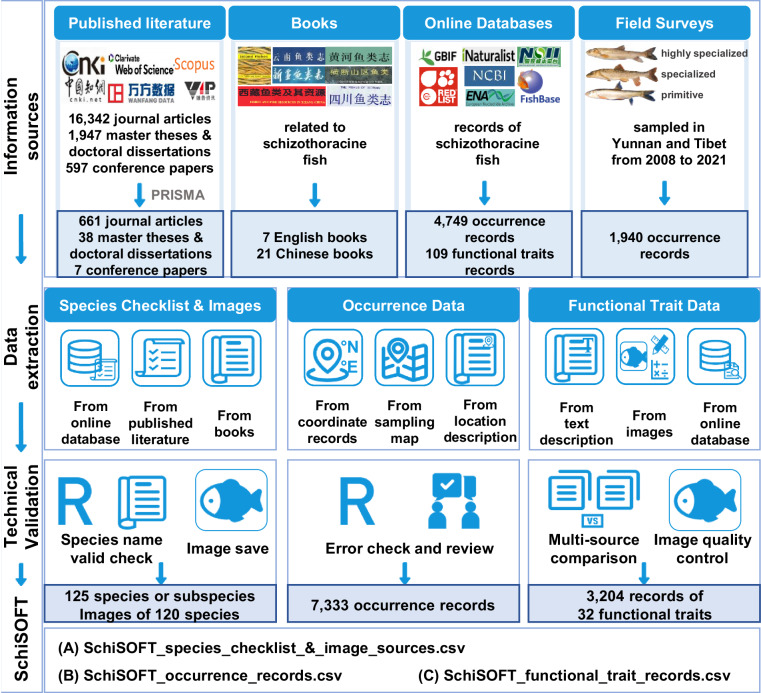
The workflow to compile the SchiSOFT dataset on species occurrences and functional traits of Schizothoracinae fish.

In this study, we introduce the SchiSOFT^33^ (Schizothoracinae fish Species Occurrences and Functional Traits) dataset, which compiled and curated data from our long-term survey records, possible online databases (e.g., FishBase and GBIF), and systematically searched literature (Fig. 1). The literature covers both that written in Chinese and English, and the publication date spans from 1842 to 2022. Details such as sampling locations, geographic coordinates, sampling dates, and functional traits (e.g., maximum body length, scale coverage, and pharyngeal teeth) were gathered, collated, and verified. The SchiSOFT^33^ presents the most comprehensive dataset of Schizothoracinae fish, including all 125 species or subspecies from the 12 genera, 7,333 occurrence records, and 3,204 records of 32 functional traits. Sampling records spanned over 180 years (1840s–2020 s). This dataset enables researchers and managers to quickly acquire specific information (e.g., distribution range, functional traits) about Schizothoracinae fish through querying corresponding fields such as scientific names, genus names. Thus, it can promote research, conservation, and management of Schizothoracinae fish diversity and resources and further ensure the goods and services they provide for both natural ecosystems and human society. It is also accessible to the public and can be used for educational activities, contributing to a deeper public understanding and awareness of the conservation of Schizothoracinae fish.

## Methods

### Information sources

The occurrence records and functional traits were primarily extracted from the following four sources: (1) published literature (e.g., journal articles, master theses, doctoral dissertations, and conference papers); (2) books (e.g., key books and ichthyographies); (3) online databases (e.g., FishBase, GBIF and National Specimen Information Infrastructure [NSII, http://www.nsii.org.cn/]); (4) field surveys over decades conducted by the authors’ research groups. We conducted a systematic literature search in multiple databases, such as the Web of Science (WoS, https://www.webofscience.com/), Scopus (https://www.scopus.com/), and the Chinese National Knowledge Infrastructure (CNKI, https://www.cnki.net/). We also searched for books and online databases (e.g., FishBase and GBIF). The search was initially conducted in October 2020 and updated in October 2022.

Our search queries were based on the names of the target fish (e.g., scientific genus names and common names). Data from the WoS and Scopus, based on titles, abstracts, and keywords, were searched using the following English search phrase: (Schizothoracinae OR schizothoracine OR Schizopygopsinae OR *Aspiorhynchus* OR *Chuanchia* OR *Diptychus* OR *Herzensteinia* OR *Gymnocypris* OR *Oxygymnocypris* OR *Platypharodon* OR *Schizothorax* OR *Schizopygopsis* OR *Schizocypris* OR *Racoma* OR *Schizothoraichthys* OR *Ptychobarbus* OR *Oreinus* OR schizothoracin OR snowtrout OR marinka OR “naked carp”). The Chinese search phrase was generally the same as the English version, searched from the CNKI, Wanfang Database (https://www.wanfangdata.com.cn/), and Weipu Database (https://qikan.cqvip.com/). After removing 8,415 duplicates through fuzzy title matching using the restricted Damerau-Levenshtein distance similarity^34^, a total of 18,886 references were retained. We then screened the titles, keywords, and abstracts of the documents returned by the search and excluded records with explicit reasons as follows: (1) reviews without sampling data; (2) river basin scale or regional aquatic field surveys with no records of schizothoracine fish occurrence data; (3) research articles that do not include field sampling or only used environment DNA methods; (4) studies with schizothoracine fish but were not identified to species level; (5) captive-bred schizothoracine fish without field sampling information. Primary research articles mentioned in review papers that potentially contain relevant data were also included to complement our reference pool (Fig. 1). The searching, screening, and filtering strictly followed the workflow of PRISMA^35^ (Preferred Reporting Items for Systematic Reviews and Meta-Analysis). Finally, we obtained data from 706 pieces of published literature, 28 books in English and Chinese, and seven online databases, including FishBase, NSII, GBIF, the European Nucleotide Archive (ENA, https://www.ebi.ac.uk/ena/), the National Center for Biotechnology Information (NCBI, https://www.ncbi.nlm.nih.gov/), iNaturalist (https://www.inaturalist.org/), IUCN Red List of Threatened Species (IUCN, https://www.iucnredlist.org/). Schizothoracinae species occurrences and photos for functional trait measurement collected during field surveys conducted by our research groups spanned fourteen years^33^ (i.e., 2008–2021). These data are clearly noted in the spreadsheet at figshare^33^.

### Data extraction

We extracted occurrence information for each species, including scientific names, georeferenced locations, and sampling times (Table 1), from the text, tables, figures, and supporting information from all the sources. To extract data from maps and other types of figures, we used the WebPlotDigitizer^36^ (Version 4.4). The occurrence records were cleaned to remove outliers, for example, those records with high spatial uncertainty, using the R package ‘*CoordinateCleaner*’^37^. Occurrence coordinates were recorded in decimal degrees (see section below ‘Technical Validation’).

**Table 1:** Descriptions of the fields used in the SchiSOFT dataset.

Field	Description
genus	genus of the species.
specializedGrade	the specialized grade of Schizothoracinae fish, including primitive grade, specialized grade, and highly specialized grade.
scientificName	scientific name of the species.
taxonomicStatus	species or subspecies.
IUCNcategory	the species IUCN Red List extinction risk, including LC (*Least Concern*), DD (*Data Deficient*), VU (*Vulnerable*), NT (*Near Threatened*), EN (*Endangered*), CR (*Critically Endangered*), EX (*Extinct*), and ‘NA’ if not available.
occurrenceID	the identification code originally provided for the occurrence record.
originalNameInSources	original species name in sources.
sampledDate	the actual sampled date, or approximate date, of the occurrence record occurred. Four characters represent the sampling year, six characters represent the sampling year and month, and eight characters represent the sampling day. If the sampling date was a period, connect with ‘_’.
longitudeX	geographical longitude in decimal degrees of the occurrence record.
latitudeY	geographical latitude in decimal degrees of the occurrence record.
remarksOfSampledDate	remarks of sampled date, if there was no sampling date recorded in the sources, we recorded the earliest available date of the sources as a substitute, such as the received date of the article, etc. ‘NA’ indicated the actual sampling date in the field ‘sampledDate’.
sourceLanguage	language of sources, including English and Chinese, ‘NA’ if sourced from field surveys.
sourceType	the source of the record, including published literature (journal articles, master theses, doctoral dissertations, and conference papers), books, online databases, and field surveys.
referencesInEnglish	the citation of references published in English, and Chinese references had been translated into English. ‘NA’ if sourced from field surveys.
referencesInChinese	the citation of references published in Chinese, ‘NA’ if sourced from field surveys or published in English.
DOIorISBN	the DOI code of published literature and the ISBN code of books, ‘NA’ if not available or sourced from field surveys.
imageType	the image type of the Schizothoracinae fish, including photo and scientific drawing, ‘NA’ if not available.
URL	the URL for the exact online database information.

The functional traits of schizothoracine fish are essential for understanding their evolutionary adaptation and responses to historical and modern environmental changes in the QTP. Our dataset encompasses 32 functional traits, which can be grouped into five categories: multi-functional (5 traits; e.g., maximum body length), trophic (16 traits; e.g., feeding habits and oral gape position), locomotion (6 traits; e.g., body elongation), life history (2 traits; e.g., fecundity), and habitat utilization (3 traits; e.g., habitat substrate) (Table 2). Maximum body length and maximum body weight data were mainly taken from FishBase and supplemented from books. Twelve commonly used ratio traits (continuous data; e.g., relative eye size and caudal fin aspect ratio) in evaluating the morphological diversity of freshwater fish^26,38–41^ were measured from specimen photos or images (i.e., scientific drawings of fish lateral views) with the assistance of ImageJ software (http://rsb.info.nih.gov/ij/index.html). The rest of the trophic traits, life history traits, and habitat utilization traits, which are mostly categorical, were extracted from text descriptions in books (e.g., *Fauna Sinica*^17^, *The Fishes of the Qinghai-Xizang Plateau*^26^, and *The Fishes of the Hengduan Mountains Region*^42^) and taxonomic research articles.

**Table 2:** Descriptions of the functional traits in the SchiSOFT dataset.

Categories	Traits name	Data type	Description of functional traits or formulas, and the sources
multi-functional	lateralLine	Categorical	lateral line complete or not complete, including ‘complete’ and ‘NA’; derived from text descriptions.
	scaleCoverage	Categorical	scale coverage of the species, including ‘A’: body with minute scales; ‘B’: body with minute scales expect venter; ‘C’: more or less minute scales on body sides and tail; ‘D’: almost entirely naked; and ‘NA’; derived from text descriptions.
	maximumBodyLength	Numerical.c	maximum body length (mm) of the species, range from 107 to 1058, ‘NA’; derived from FishBase or books, partially used the ratio of body length to total length to calculate if there was only total length data.
	maximumBodyWeight	Numerical.c	maximum body weight (kg) of the species, range from 0.05 to 23, ‘NA’; derived from FishBase or book descriptions.
	verticalEyePosition	Numerical.c	eye height/body depth, range from 0.25 to 0.64, ‘NA’ if not available; derived from image measurements.
trophic	dorsalRay	Categorical	last simple dorsal ray strong or soft, including ‘strong’, ‘soft’, and ‘NA’; derived from text descriptions.
	feedingHabits	Categorical	feeding habits of the species, including ‘carnivores’, ‘omnivores-invertebrate’ (omnivorous diet and prefers invertebrates), ‘omnivores-periphytic algae’ (omnivorous diet and prefers periphytic algae), and ‘NA’; derived from text descriptions.
	lowerJawHorny	Categorical	lower jaw with or without a horny layer, including ‘with horny’, ‘without horny’, and ‘NA’; derived from text descriptions.
	lowerTipPapillne	Categorical	lower tip with or without papillne, including ‘with papillne’, ‘without papillne’, and ‘NA’; derived from text descriptions.
	mouthPosition	Categorical	mouth position of the species, including ‘inferior’, ‘terminal’, ‘inferior or terminal’, and ‘NA’; derived from text descriptions.
	mouthShape	Categorical	mouth shape, including ‘curve’, ‘horizontal’, ‘oblique’, ‘curve or horizontal’, ‘horizontal or oblique’, and ‘NA’; derived from text descriptions.
	pharyngealTeethFormation	Categorical	pharyngeal teeth formation, including ‘2,3,4/4,3,2’, ‘2,3,5/5,3,2’, ‘3,4/4,3’, ‘4/4’, and ‘NA’; derived from text descriptions, when there are multiple cases of pharyngeal teeth formation in a species, recorded most common formation.
	postlabialGroove	Categorical	postlabial groove interrupted or continuous, including ‘continuous’, ‘interrupted’, and ‘NA’; derived from text descriptions.
	eyeSize	Numerical.c	eye diameter/head depth, range from 0.06 to 0.4; derived from image measurements, ‘NA’ if not available.
	intestineLength	Numerical.c	intestine length/body length, range from 0.75 to 11.7; derived from text descriptions, ‘NA’ if not available.
	maxillaBarbLength	Numerical.c	maxilla barbs length/head length, range from 0.01 to 0.5, ‘0’ for no maxilla barbs, and ‘NA’ if not available; derived from image measurements.
	maxillaryLength	Numerical.c	maxillary jaw length/head depth, range from 0.14 to 0.59, ‘NA’ if not available; derived from image measurements.
	oralGapePosition	Numerical.c	mouth height/body depth, range from 0.1 to 0.53, ‘NA’ if not available; derived from image measurements.
	rectalBarbLength	Numerical.c	rectal barbs length/head length, range from 0.003 to 0.73, ‘0’ for no rectal barbs, and ‘NA’ if not available; derived from image measurements.
	barbPairs	Numerical.d	number of pairs barbs, including ‘0’, ‘1’, ‘2’, and ‘NA’; derived from text descriptions.
	pharyngealTeethRows	Numerical.d	the minimal number of pharyngeal teeth rows for each species, including ‘1’, ‘2’, ‘3’, and ‘NA’; derived from text descriptions.
locomotion	bodyElongation	Numerical.c	body length/body depth, range from 3.5 to 6.5, ‘NA’ if not available; derived from image measurements.
	bodyLateralShape	Numerical.c	head depth/body depth, range from 0.5 to 0.85, ‘NA’ if not available; derived from image measurements.
	caudalFinAspectRatio	Numerical.c	caudal fin depth^2/caudal fin square, range from 0.96 to 2.87, ‘NA’ if not available; derived from image measurements.
	caudalPeduncleThrottling	Numerical.c	caudal fin depth/caudal peduncle depth, range from 1.42 to 4.3, ‘NA’ if not available; derived from image measurements.
	pectoralFinPosition	Numerical.c	pectoral fin position/body depth, range from 0.12 to 0.40, ‘NA’ if not available; derived from image measurements.
	pectoralFinSize	Numerical.c	pectoral fin length/body length, range from 0.12 to 0.21, ‘NA’ if not available; derived from image measurements.
life history	startSpawningSeason	Categorical	the season start spawning of the species, including ‘winter’, ‘spring’, ‘summer’, and ‘NA’; derived from text descriptions.
	fecundity	Numerical.c	the number of eggs laid by a female fish during the spawning season, range from 1,550 to 190,000, and ‘NA’ if not available; derived from text descriptions.
habitat utilization	substrate	Categorical	preferred habitat substrate of the species, including ‘mud’, ‘sand’, ‘gravel’, ‘pebbles’, ‘mixed substrate’, and ‘NA’; derived from text descriptions.
	habitatFlow	Categorical	preferred waterflow velocity of the species, including ‘slow flow’, ‘rapid flow’, and ‘NA’; derived from text descriptions.
	waterbody	Categorical	preferred waterbody type of the species, including ‘lake’, ‘river’, ‘lake and river’, and ‘NA’; derived from text descriptions.

Categorical indicated the categorical data type. ‘Numerical.d’ indicated the numerically discrete data type, and ‘Numerical.c’ indicated the numerically continuous data type.

### Species, taxonomy, and status

The scientific names of all Schizothoracinae fish included in the dataset have all been thoroughly checked for typing errors and misspellings. To avoid including invalid species and synonyms, we verified the validity of each species according to FishBase, using R package ‘*rfishbase*’^43^. For the species or subspecies that was not matched, it would be searched again in Eschmeyer’s Catalog of Fishes. For the subspecies that were designated as subspecies in *Fauna Sinica*^17^, but were not identified in FishBase and Eschmeyer’s Catalog of Fishes, they were listed as valid subspecies in our dataset. The final standardized species list has 125 valid species (i.e., 98 species and 27 subspecies) (see section below ‘Technical Validation’).

## Data Records

**Fig. 2:**
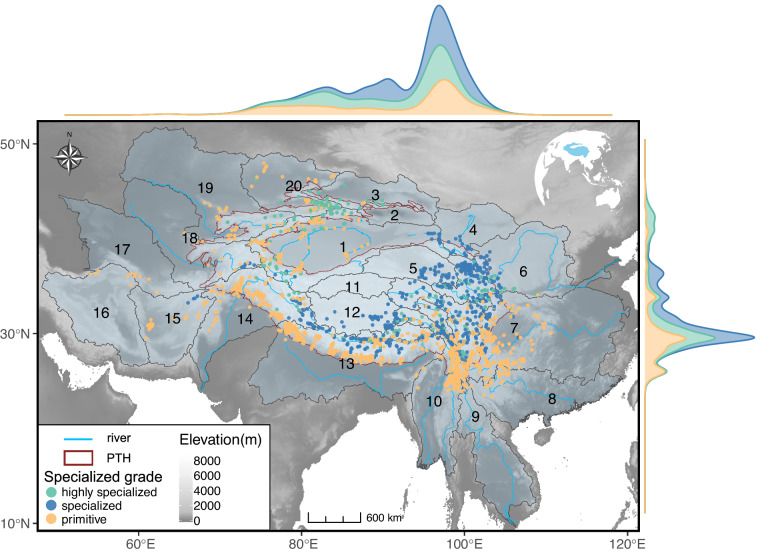
Occurrence points of the Schizothoracinae fish by specialized grades at the global scale in the SchiSOFT dataset. The lines in blue show the main river around the Qinghai-Tibetan Plateau (QTP), and the lines in red show the boundary of the Pan-Tibetan Highlands (PTH). The 20 regions are referred to as Level 3 in HydroBASINS (https://www.hydrosheds.org/products/hydrobasins). 1: Tarim, 2: Turfan, 3: Dzungaria, 4: Hexi, 5: Qaidan, 6: Yellow River, 7: Yangtze River, 8: Xi Jiang, 9: Lancang-Mekong, 10: Salween-Irrawaddy, 11: Hoh xil, 12: Inner Tibetan Plateau, 13: Brahmaputra-Ganges, 14: Indus, 15: Helmand-Sistan, 16: Kavir and Lut Deserts, 17: Amu Darya WEST, 18: Amu Darya EAST, 19: Syr Darya, 20: Balkash-Alakul.

Our final dataset^33^ has been deposited at figshare and can be downloaded from 10.6084/m9.figshare.24638538.v1. It includes a total of 7,333 occurrence records, and the total number of functional trait records is 3,204 (Fig. 2, Table 2). Among them, 3,876, 844, and 673 occurrence records were from 706 publications, 28 books, and seven online databases, respectively. The remaining 1,940 records were from field surveys conducted by our research groups over fourteen years (2008–2021). And the functional trait mean data completeness was 80.1%. Among them, 1,424 records were extracted from images, 109 records were extracted from online databases, and 1,671 records were obtained from text descriptions of published documents.

Occurrences and functional traits were recorded according to uniform standards. The dataset^33^ was organized into three CSV-format files. (A), “SchiSOFT_species_checklist_&_image_sources.csv”, includes genus name, specialized grade, scientific name, taxonomic status, IUCN Red List extinction risk, image type, source type, references in English, and URL. (B), “SchiSOFT_occurrence_records.csv”, includes the genus name, scientific name, the original species name in the sources, taxonomic status, the sampled date, and remarks of the sampled date, decimal latitude and latitude, the source language, source type, references in English, references in Chinese, DOI or ISBN code. If there was no sampled date recorded in the sources, we recorded the received date, accepted date, and published date of the source document as a substitute, if available. The citations of Chinese publications had also been translated into English, recorded in ‘referenceInChinese’ and ‘referenceInEnglish’, respectively. (C), “SchiSOFT_functional_trait_records.csv”, includes the scientific name and 32 functional traits records (Table 2).

(A), “SchiSOFT_species_checklist_&_image_sources.csv”, contains the checklist of 125 species or subspecies of schizothoracine fish, including fields: ‘genus’, ‘specializedGrade’, ‘scientificName’, ‘taxonomicStatus’, ‘IUCNcategory’, ‘imageType’, ‘sourceType’, ‘referenceInEnglish’, and ‘URL’ (Table 1).

(B), “SchiSOFT_occurrence_records.csv”, contains the species list and geographic coordinates, including 15 fields: ‘occurrenceID’, ‘genus’, ‘scientificName’, ‘originalNameInSources’, ‘taxonomicStatus’, ‘sampledDate’, ‘longitudeX’, ‘latitudeY’, ‘remarksOfSampledDate’, ‘sourceLanguage’, ‘sourceType’, ‘referenceInEnglish’, ‘referenceInChinese’, ‘URL’, and ‘DOIorISBN’ (Table 1).

(C), “SchiSOFT_functional_trait_records.csv”, contains functional trait data of Schizothoracinae fish, including 34 fields: ‘genus’, ‘scientificName’, ‘lateralLine’, ‘scaleCoverage’, ‘maximumBodyLength’, ‘maximumBodyWeight’, ‘verticalEyePosition’, ‘dorsalRay’, ‘feedingHabits’, ‘lowerJawHorny’, ‘lowerTipPapillne’, ‘mouthPosition’, ‘mouthShape’, ‘pharyngealTeethFormation’, ‘postlabialGroove’, ‘eyeSize’, ‘intestineLength’, ‘maxillaBarbLength’, ‘maxillaryLength’, ‘oralGapePosition’, ‘rectalBarbLength’, ‘barbPairs’, ‘pharyngealTeethRows’, ‘bodyElongation’, ‘bodyLateralShape’, ‘caudalFinAspectRatio’, ‘caudalPeduncleThrottling’, ‘pectoralFinPosition’, ‘pectoralFinSize’, ‘startSpawningSeason’, ‘fecundity’, ‘substrate’, ‘habitatFlow’, and ‘waterbody’ (Table 2).

## Technical Validation

### Taxonomic and status validation

Each original species name was compared to the list of valid species names in FishBase, Eschmeyer’s Catalog of Fishes, *Fauna Sinica*^17^, *The Fishes of the Qinghai-Xizang Plateau*^26^ or *Xinjiang Ichthyology*^44^ to ensure the identification validity provided by the information source. In the column ‘originalNameInSources’, the original scientific names in sources were recorded fully for checks and verifications. The R package ‘*rfishbase*’^43^ was used to do a batch search and matching for species names. For the species or subspecies that was not matched, it would be searched again in Eschmeyer’s Catalog of Fishes. There were 125 valid species names (98 species and 27 subspecies), including the subspecies designated as subspecies in *Fauna Sinica*^17^ but identified as species in FishBase or Eschmeyer’s Catalog of Fishes. In addition, our dataset removed misidentified species.

### Species distribution validation

Sampling points with accurate latitude and longitude in all sources were recorded directly in decimal degrees. To extract data from the sampling maps, we used the WebPlotDigitizer^36^ (Version 4.4). The coordinates of occurrence records with exact sampling point descriptions were located using Google Earth (https://earth.google.com/). For occurrence data recorded at a coarse spatial resolution (e.g., villages, towns, and even counties with relatively extensive coverage), their coordinates were determined by combining location names and the sampled rivers or streams. The native distribution range of the species and transportation accessibility were also used as supporting information. Generally, these points were within a 10-kilometer radius from the area centre. In cases where the area was too large with a complex river network, we discarded those occurrence records directly. Unclear sampling ranges described in the text were eliminated. For example, only river basins or sub-basins were described without information on administrative boundaries; sub-basins were reported with administrative boundaries placed at the provincial or city level. All sampling points were fixed to the river network based on the Hydrography90m^45^ using the ‘*NEAR*’ function in the software ArcGIS (Version 10.4). The occurrence records were cleaned to remove outliers and records with high spatial uncertainty using the R package ‘*CoordinateCleaner*’^37^, the cleared geographic coordinates had been rechecked manually.

Then, the geographic coordinates of the occurrence points were checked and validated with the river name or the administrative district that was described in the sources, such as the county, town, and village names. In the event of a mismatch, the coordinates were removed from the dataset after double-checking. Records from the online databases, books, master theses, doctoral dissertations, and articles may share field sampling; in this case, duplicated occurrence records were removed. Finally, the distribution basins of 125 species or subspecies were checked and reviewed to eliminate non-natural distributions induced by religious release activities or artificial enrichment releases. For the endangered species or threatened species in our dataset^33^, we have kept geographic coordinates rounded to 0.1 degree of the latitude and longitude^46^. The IUCN Red List status of these species includes *Critically Endangered* (CR), *Endangered* (EN), *Extinct in the Wild* (EW), and *Extinct* (EX). Detailed data of these species can be supplied to researchers on request. All the occurrence records were given an ID number, and the ‘occurrenceID’ is unique and can be checked in “SchiSOFT_occurrence_records.csv”^33^.

### Functional traits data validation

For the functional traits, which were taken from books and published literature, each species of schizothoracine fish gathered from two or more sources as much as possible to avoid incorrect data. We compared and checked the data for the same species recorded from text descriptions in different sources to see if there were differences or deviations. Where differences or deviations existed, we added as many sources as possible about the species, and the same or similar descriptions in most of the literature were adopted. If the recorded data from the documents was an interval value, the median value was recorded in our dataset^33^. The maximum body length and maximum body weight data were mainly taken from FishBase and supplemented by books and published literature. In the case where there was only total length data without body length data, we used the ratio of body length to total length extracted from the images to calculate the body length.

The most ratio traits were measured from specimen images (i.e., photos or scientific drawings of fish lateral views). Scientific drawings were primarily sourced from *Fauna Sinica*^17^, *The Fishes of the Qinghai-Xizang Plateau*^26^ and *Xinjiang Ichthyology*^44^, and specimen photos were mostly downloaded from FishBase, supplemented by journal articles and museum photos. When several images with a lateral view were available for a species, measurements were taken on the one with the best quality. Photos of museum specimens were also used only if they could provide a morphological representation of the fish species. The quality of the photos did not allow for the measurement of all morphological traits of all species due to improper body positioning and specimen distortion. All those doubtful measurements were scrapped and recorded as ‘NA’. The sources of photos and scientific drawings were recorded in our dataset^33^, “SchiSOFT_species_checklist_&_image_sources.csv”.

## Usage Notes

Based on published literature, books, online databases, and field surveys, we collected a full species and image sources list, occurrence data, and detailed functional traits data for Schizothoracinae fish. The dataset^33^ is obviously not complete and conclusive, and we aim to support the dataset with regular updates, ideally with biannual or triennial steps, depending on the available resources. Three main factors will be considered in future updates: (1) new or previously unavailable data sources (e.g., investigated reports of Sichuan and Qinghai Provinces) with species lists or records for additional drainage basins or drainage basins already present in the dataset; (2) the distribution of newly described species; and (3) nomenclature changes in the taxonomic classification. This collection not only offers high-resolution occurrence data but also presents intricate details regarding the functional traits of Schizothoracinae fish.

The dataset limitations are manifested in: (1) the distribution of Schizothoracinae fish in the high-elevation, low-oxygen areas of the QTP and its surroundings, as acquiring specimens is challenging due to accessibility limits and sampling bias; (2) Schizothoracinae-related documents written in languages other than English and Chinese are not included in this dataset, and the grey documents are also not included.

Our dataset serves multiple purposes, making it invaluable for various scientific inquiries. First and foremost, it provides a solid foundation for historical biogeographical research centered on Schizothoracinae fish, particularly in the context of the QTP uplift. Furthermore, the occurrence data can be harnessed to predict shifts in species distribution by employing ecological niche models or species distribution models to improve the future protected area management paired with high-resolution geographic or climatic data. The functional traits data play an important role in delving even deeper into the distribution dynamics of Schizothoracinae and the underlying factors contributing to these variations.

## Data Availability

The R code used to perform batch searching, matching, and checking of species names from the original species names using the R package ‘*rfishbase*’^43^ and to clean coordinates using the R package ‘*CoordinateCleaner*’^37^ (“SchiSOFT_check_clean.R”) is available at 10.6084/m9.figshare.24638538.v1.
